# Supplementary material for: Collaborative research to support urban agriculture in the face of change: The case of the Sumida watercress farm on O‘ahu
Source: PLoS One. 2020 Jul 23;15(7):e0235661. doi: 10.1371/journal.pone.0235661 (PMC7377374; doi:10.1371/journal.pone.0235661)
Supplement: S1 Table — (PDF) [file pone.0235661.s001.pdf]

Supplemental Table 1. Primer sets used for quantitative PCR.

Primer name	Sequence	Reference
Bacterial 16S (*16S*)	Bact1369F- CGGTGAATACGTTCYCGG<br>Prok1541R- AAGGAGGTGATCC RGCCGCA	Suzuki et al., 2001
Nitrogenase (*nifH*)	nifH_F - TGCGAYCCSAARGCBGACTC<br>nifH_R - ATSGCCATCATYTCRCCGGA	Poly et al., 2001
Nitrite reductase (*nirS*)	nirS_F - TACCACCCSGARCCGCGCGT<br>nirS_R - GCCGCCGTCRTGVAGGAA	Chon et al., 2011
Bacterial Anammox (*amoA*)	amoA_F - GGGGTTTCTACTGGTGGT<br>amoA_R - CCCCTCKGSAAAGCCTTCTTC	Zhang et al., 2012

Chon, K, Chang, J.S., Lee, E., Lee, J., Ryu, J., and Cho, J. (2011) Abundance of denitrifying genes coding for nitrate (*narG*), nitrite (*nirS*), and nitrous oxide (*nosZ*), reductases in estuarine versus wastewater effluent-fed constructed wetlands. Ecol Engr 37:64-69.

Poly, F., Monrozier, L.J., and Bally R. (2001) Improvement in the RFLP procedure for studying the diversity of *nifH* genes in communities of nitrogen fixers in soil. Res Micro 152:95-103

Suzuki, M., Nakagawa, Y., Harayama, S., and Yamamoto, S. (2001) Phylogenetic analysis and taxonomic study of marine Cytophaga-like bacteria: Proposal for *Tenacibaculum* gen. nov. with *Tenacibacuum maritimum* comb. Nov. and *Tenacibaculum ovolyticum* comb. Nov., and description of *Tenacibaculum mesophilum* sp. Nov. and Tenacibaculum amylolyticum sp. Nov. Int J Syst Evol Microbiol 51: 1639-1652.

Zhang, L.M., Hang-Wei, H. Shen, J.P, and He, J.Z. (2012) Ammonia-oxidizing archaea have more important role than ammonia-oxidizing bacteria in ammonia oxidation of strongly acidic soils. ISME 6:1032-1045.
