# Supplementary material for: Collaborative research to support urban agriculture in the face of change: The case of the Sumida watercress farm on O‘ahu
Source: PLoS One. 2020 Jul 23;15(7):e0235661. doi: 10.1371/journal.pone.0235661 (PMC7377374; doi:10.1371/journal.pone.0235661)
Supplement: S2 Table — Abbreviations in the table include: Radon, Rn; Salinity, Sal; Specific Conductivity, SPC; Dissolved Oxygen, DO; Temperature, Temp; Caffeine, Caf; ethynylestradiol, EE2; Atrazine, Atr; and Dicholor-diphenyl-trichloroethane and its degradation product DDE, DDT. Stable isotopes of oxygen, δ18O; hydrogen, δD; Standard deviation of both isotopes, SD δ18O and SD δD; Total dissolved nitrogen, Total N; Total dissolved phosphorus, Total P; dissolved inorganic P, P; Nitrite+Nitrate, N+N; Isotopes of 15N- as NO3-, δ15N-NO3-, and oxygen as -NO3-, δ18O-NO3-. Detection limits, dl include: Caffeine dl = 150 ng/L; Carbamazepine dl = 25 ng/L; EE2 dl = 50 ng/L; Atrazine dl = 50 ng/L;, DDT dl = 0.6 ng/L. (PDF) [file pone.0235661.s002.pdf]

Supplemental Table 2. Chemical parameters measured in selected springs at Sumida farm. Rn, Radon; Sal, Salinity; Specific Conductivity, SPC; DO, Dissolved Oxygen; Temp, Temperature; Caffeine, Caf; ethynylestradiol, EE2; Atrazine, Atr, Dicholor-diphenyl-trichloroethane, DDT. Detection limits, dl include; Caffeine dl=150 ng/L; Carbamazepine dl=25 ng/L; EE2 dl=50 ng/L; Atrazine dl=50 ng/L; DDT dl=0.6 ng/L.

Site	Date	Rn (dpm/L)	Sal	SPC	DO (%)	DO (mg/L)	Temp (˚C)	Caf (ng/L)	Car (ng/L)	EE2 (ng/L)	Atr (ng/L)	DDT (ng/ml)
Middle Big Spring	8/14/18	282.1	1.24	2.409	113.8	10.05	20.6	<dl	91			
	2/12/19	262	1.4	2.66	92.7	8.35	20.7		181	<dl	40	2.4
Middle Covered Spring	2/12/19	272	1.1	2.23	92.0	8.22	20.5		161	<dl	40	2.1
Mauka Spring	8/14/18	237.5	0.30	0.627	116.7	10.47	20.4	<dl				
	2/12/19	211	0.30	0.517	93.7	8.43	20.3		22	<dl	30	2.4
Spring N	2/12/19	154	0.38	0.77	92.8	8.07	21.1		81	<dl	30	2.0
Spring C	2/12/19	141	0.38	0.77	96.2	8.48	21.3		130	<dl	20	1.8
Spring S	2/12/19	189	0.35	0.72	81	7.34	21.3		36	<dl	30	2.4

Supplemental Table 2 continued. Stable isotopes of oxygen, δ18O; hydrogen, δD; Standard deviation, SD δ18O and SD δD; Total dissolved nitrogen, Total N; Total dissolved phosphorus, Total P; dissolved inorganic P, P; Nitrite+Nitrate, N+N; Isotopes of 15N- as NO3-, δ15N-NO3-, and oxygen as -NO3-, δ18O-NO3-.

Site	Date	δ18O (‰)	δD (‰)	SD δ18O (‰)	SD δD (‰)	Total N (uM)	Total P (uM)	P (uM)	Silicate (uM)	N+N (uM)	NH_4 (uM)	δ15N-NO3- (‰)	δ18O-NO3- (‰)
Middle Big Spring	8/14/18	-3.1	-10.5	0.02	0.18								
	2/12/19	-3.0	-10.4	0.02	0.20	28.78	1.79	1.39	845.55	20.59	<0.02	5.39	4.74
Middle Covered Spring	2/12/19	-3.1	-10.1	0.01	0.12	29.29	1.63	1.24	849.39	21.66	<0.02	5.21	4.59
Mauka Spring	8/14/18	-3.1	-10.0	0.02	0.09								
	2/12/19	-3.1	-9.9	0.03	0.10	32.52	2.82	2.47	926.51	25.14	<0.02	5.43	4.20
Spring N	2/12/19	-3.0	-9.7	0.04	0.17	36.78	2.80	2.45	915.95	26.25	<0.02		
Spring C	2/12/19	-3.0	-9.6	0.01	0.05	36.64	2.89	2.56	912.91	25.40	<0.02	6.01	4.48
Spring S	2/12/19	-3.1	-9.7	0.02	0.09	33.75	2.77	2.42	925.87	22.66	<0.02	6.29	5.11
