# Supplementary material for: Collaborative research to support urban agriculture in the face of change: The case of the Sumida watercress farm on O‘ahu
Source: PLoS One. 2020 Jul 23;15(7):e0235661. doi: 10.1371/journal.pone.0235661 (PMC7377374; doi:10.1371/journal.pone.0235661)

Supplemental Figure 1. Sumida Farm and surrounding groundwater pumping wells in the Waimalu groundwater management unit (black dot indicates location of Sumida Farm; red dots denote pumping wells strongly weighted in the inverse-distance weighted sum; purple dots denote pumping wells weakly weighted in the inverse-distance weighted sum).

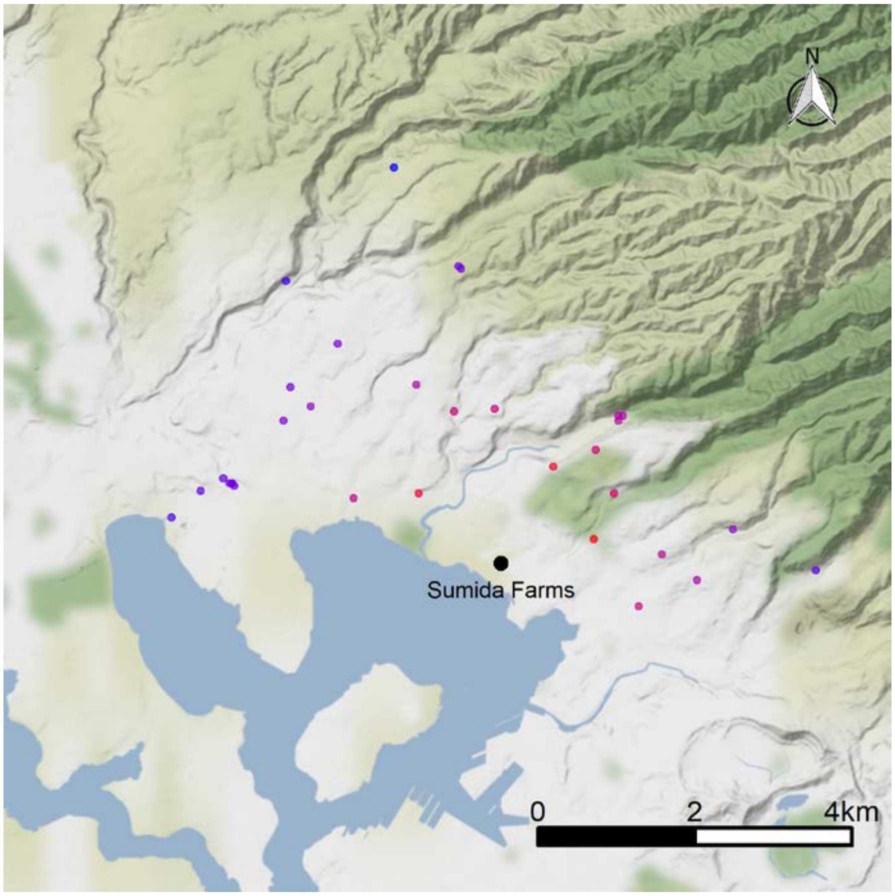

Supplement: S1 Fig — (PDF) [file pone.0235661.s003.pdf]
