# Supplementary material for: Collaborative research to support urban agriculture in the face of change: The case of the Sumida watercress farm on O‘ahu
Source: PLoS One. 2020 Jul 23;15(7):e0235661. doi: 10.1371/journal.pone.0235661 (PMC7377374; doi:10.1371/journal.pone.0235661)

Supplemental Figure 2. Standard curves of 16S, amoA, nifH, and nirS qPCR assays acquired by plotting gene copy number (log copies) by threshold cycle (Ct). Equations for 16S: $y=-3.4621x + 38.896$; amoA: $y=-3.5148x + 47.645$; nifH: $y=-3.4341x + 36.174$; and nirS: $y=-3.3436x + 43.568$.

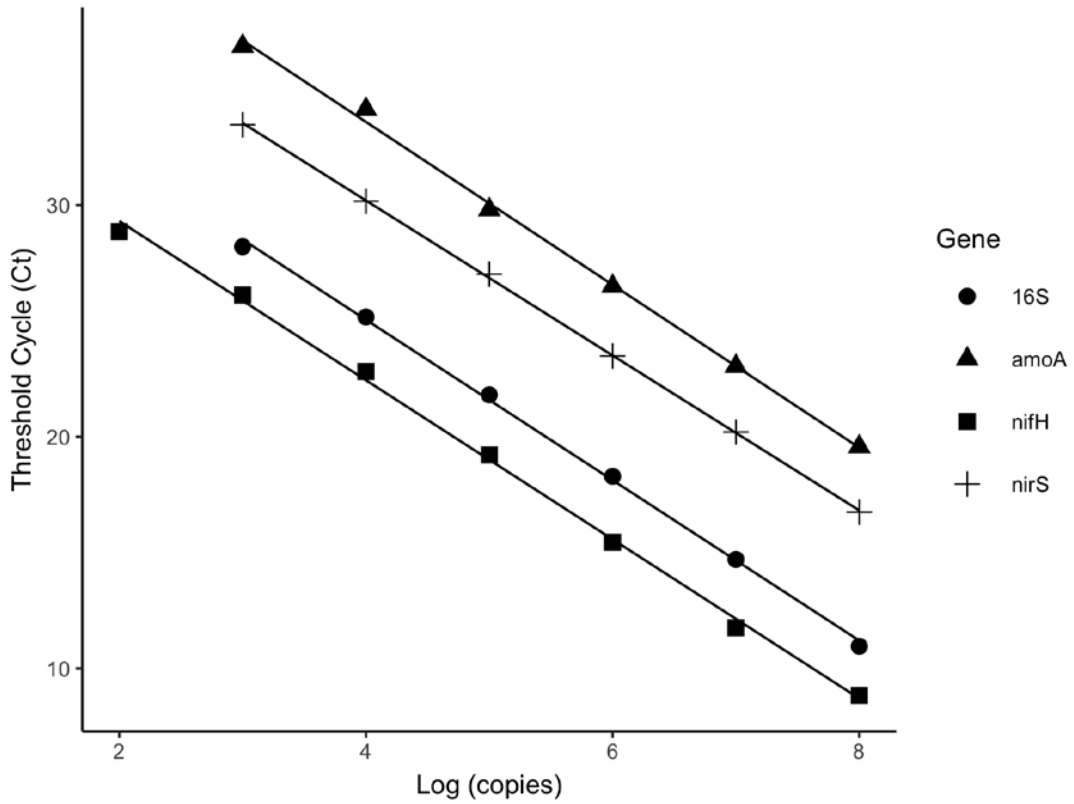

Supplement: S2 Fig — Equations for 16S: y = -3.4621x + 38.896; amoA: y = -3.5148x + 47.645; nifH: y = -3.4341x + 36.174; and nirS: y = -3.3436x + 43.568. (PDF) [file pone.0235661.s004.pdf]
