# Supplementary figures and images for: Collaborative research to support urban agriculture in the face of change: The case of the Sumida watercress farm on O‘ahu
Source: PLoS One. 2020 Jul 23;15(7):e0235661. doi: 10.1371/journal.pone.0235661 (PMC7377374; doi:10.1371/journal.pone.0235661)

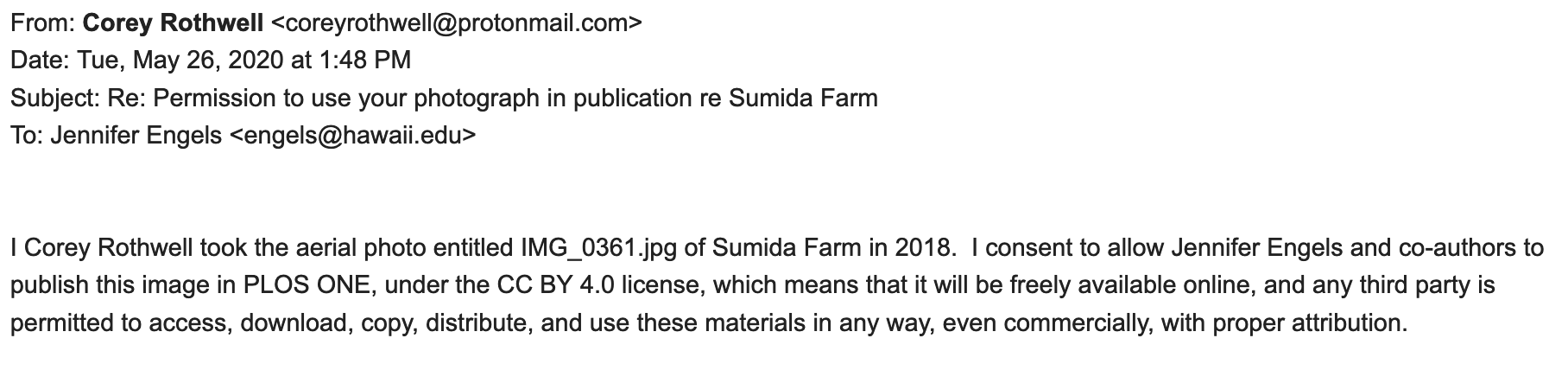

Supplement: S3 Fig — (PNG) [file pone.0235661.s005.png]
