# Supplementary material for: Collaborative research to support urban agriculture in the face of change: The case of the Sumida watercress farm on O‘ahu
Source: PLoS One. 2020 Jul 23;15(7):e0235661. doi: 10.1371/journal.pone.0235661 (PMC7377374; doi:10.1371/journal.pone.0235661)
Supplement: Attachment — Submitted filename: 01 - 040420 response to reviewers .docx [file pone.0235661.s007.docx]

Jennifer Engels

Hawai‘i Institute of Geophysics and Planetology

University of Hawai‘i at Mānoa

1680 East-West Road

Honolulu, HI 96822

engels@hawaii.edu

April 3, 2020

Dear Dr. Duan:

We thank you and the two reviewers for your constructive and helpful feedback to our manuscript, now entitled “Collaborative research to support urban agriculture in the face of change: the case of the Sumida watercress farm on Oʻahu” by Jennifer Engels, Sheree Watson, Henrietta Dulai, Kimberly Burnett, Christopher Wada, ‘Ano‘ilani Aga, Nathan DeMaagd, John McHugh, Barbara Sumida, and Leah Bremer. We have carefully considered each recommendation and have substantially improved the paper. The revised manuscript is now a compelling case study of the supports and challenges for urban agriculture in a changing environment.

Below are the editor and reviewer comments (in blue) and our responses (in black).

Thank you for submitting your manuscript to PLOS ONE. After careful consideration, we feel that it has merit but does not fully meet PLOS ONE’s publication criteria as it currently stands. Therefore, we invite you to submit a revised version of the manuscript that addresses the points raised during the review process.

Please carefully deal with the comments from the second reviewer.

We thank the editors and reviewers for the helpful comments. We have carefully considered each comment and revised the manuscript accordingly. We now feel this is well suited for publication in PLOS ONE as part of the urban ecosystems special issue.

We would appreciate receiving your revised manuscript by April 3, 2020. When you are ready to submit your revision, log on to <https://www.editorialmanager.com/pone/> and select the 'Submissions Needing Revision' folder to locate your manuscript file.

If you would like to make changes to your financial disclosure, please include your updated statement in your cover letter.

Please see our updated financial disclosure statement in the last paragraph of the new cover letter.

To enhance the reproducibility of your results, we recommend that if applicable you deposit your laboratory protocols in [protocols.io](http://protocols.io), where a protocol can be assigned its own identifier (DOI) such that it can be cited independently in the future. For instructions see: <http://journals.plos.org/plosone/s/submission-guidelines#loc-laboratory-protocols>

Our laboratory protocols are not novel. They are all previously published, and we referenced the kits and the companies where the kits were purchased which include standard protocols/instructions in the **Methods** descriptions. **Supplementary information** Tables S1, and Tables S2 provide data needed to replicate qPCR methods, standards data to assure efficiencies reported in the manuscript, and the geochemistry data as reported in the manuscript.

Please include the following items when submitting your revised manuscript:

- A rebuttal letter that responds to each point raised by the academic editor and reviewer(s). This letter should be uploaded as separate file and labeled 'Response to Reviewers'.
- A marked-up copy of your manuscript that highlights changes made to the original version. This file should be uploaded as separate file and labeled 'Revised Manuscript with Track Changes'.
- An unmarked version of your revised paper without tracked changes. This file should be uploaded as separate file and labeled 'Manuscript'.

Done

Please note while forming your response, if your article is accepted, you may have the opportunity to make the peer review history publicly available. The record will include editor decision letters (with reviews) and your responses to reviewer comments. If eligible, we will contact you to opt in or out.

We look forward to receiving your revised manuscript.

Kind regards,

Weili Duan, Ph.D
Academic Editor
PLOS ONE


[Note: HTML markup is below. Please do not edit.]

Reviewers' comments:

Reviewer #1: Comments have been addressed, however the manuscript is still not concise enough for a journal-standard. Authors should flash out better why the paper is important and what contribution this is bringing.

Response: We adjusted the framing of the paper based on Reviewer 2’s comments (please see below), which also addresses Reviewer 1’s suggestion to better describe the contribution of the paper. To address a concern raised by Reviewer 2 we added an analysis of local and national press showing the community value of Sumida farm, but nonetheless managed to reduce the total word count by 1000 words to make the paper more concise.

Reviewer #2: Major Items:
Thanks to the authors for revising their submission in response to reviewers’ comments on the earlier version of the paper.
Overall, and given the responses to reviewers’ comments, I wonder if it would have been much clearer to explicitly frame this paper in terms of the four basic ecosystems services (provisioning, regulating, supporting and socio-cultural) if the intention was to deploy a quant-qual methodology to show the multiple urban ecosystem services values of the Sumida Farm landscape. A table showing how these four ecosystems services are manifested in this specific case would be useful. Having abandoning the use of “resilience” as a framing concept in favour of urban ecosystems services such clarity in presentation becomes even more urgent.

Response: Thank you for this suggestion. We adjusted the framing to focus both on historical challenges, as well as the current and future multiple benefits provided by the farm. We specifically focus on crop yield (provisioning), nutrient retention and cycling (regulating and supporting), and multiple social and cultural services. We reframed the abstract, introduction, Table 1, discussion and conclusions, and the manuscript sub-headings to reflect this theme, which we agree is a compelling way to slightly shift our framing.

We also reframed question 3 to focus specifically on the additional socio-cultural benefits provided by the farm.

3. What additional socio-cultural benefits are provided by the farm to the Sumida family and surrounding community?

I note a few additional conceptual, theoretical and epistemological challenges in my comments below.
Lines 162, 314, 568, etc. reference is made to “public trust” doctrine….the implication here is that the state is not necessarily exercising its public trust obligations in surface and ground water resources, but not enough is said about what the state actually does do in order to assess this statement. For example, if the state manages some form of licencing and regulatory oversight over water abstraction, even if wholly productionist, then it could argue that these actions discharge its public trust obligations as it understands them. A difficult arises if you, in the course of your analysis, want to argue that other water or land values related to the public trust are not being respected – but then you have to be very clear about what these may be and why they are deserving of inclusion, who says so and under what conditions? The issue of “public trust” (and its putative abrogation) would be much more compelling if there were community voices challenging the hegemonic framing of public trust in groundwater management, but this does not seem to be present. Either this issue needs to be developed so that is it clearly germane to the analysis presented, or it should be removed entirely.

Response: Thank you for pointing this out. The public trust doctrine is a commonly discussed policy related to water management in Hawai’i, but we recognize the reviewer’s point that this is distracting and complex for a beyond-Hawaiʻi audience. We removed this discussion throughout the paper to better focus on historical challenges as well as multiple current and future benefits the farm provides.

L307 you say that the watercress farm “was of great value to the Sumida family and surrounding community.” – you are going to have to show how this works in the current analysis, or what you will have is a study in two parts: part 1 looking at the quantitative relations between output and a number of environmental variables on the one hand, and in part 2 a situating of the farm within Sumida family history since the 1930s and earlier favourable mention of the site and its springs in the Hawaiian language newspapers of the 19th and early 20th centuries. To really make the point about the multiple urban ecosystems values attaching to the farm, specifically, you would presumably need some sort of data from the contemporary community expressing a positive valorisation of the farm for a number of non-productivist related reasons. For example, in Line 158 you say “School and other community groups visit the farm and hear a historically common, yet currently rare story of multi-generational farming and the links between spring water and food systems.” This is good, but you need to go further and show that this story is somehow linked to broader, presumably socio-cultural, valorisations of the farm and its activities. Given the Hawaiian context, this would be easier if the crop was somehow traditional or indigenous. Do you have quotes or other data from non-Sumida sources showing that the farm is thus valued within the broader community?

Response: We agree and now have drawn on articles from community newspapers and national press that point to the value of Sumida farm, including for local food production, heritage value, and contribution to local culture. While watercress is not an indigenous crop, it has a long tradition in Hawaiʻi, and is very much valued locally in Hawaiʻi by a diversity of ethnic groups. In this way, while not introduced by Polynesians, watercress is a traditional crop in the sense that it has been cultivated in Hawaiʻi for nearly a century, prior to large plantations.

Linked to the above, in L645 you say “the Sumidas’ role in the co-production of knowledge described in the current study allows them to draw on data from different fields to better understand how their crop yields can be optimized going forward.” But I am not sure that I see “co-production” emerging out of the qualitative dataset, which seems to be comprised of semi-structured interviews with the Sumidas and a study of Hawaiian language newspapers. “Co-production” is usually invoked when there are multiple “knowledges” applying to the same land (e.g. productivist and non-productivist) and there is some process of reconciling them through negotiation or brokering. Multiple knowledges/values in and of themselves are not enough to establish “co-production”. Your reframed third research question (noted in your response to reviewers) “From the perspective of the farming family, and current and historical narratives, what additional benefits are associated with Sumida Farm and Kalauao Spring that may also influence the farm’s persistence over time?” may not be, by itself, enough to establish “co-production” of knowledge.

Response: While we see our work as co-production as we worked with the farm to create research questions and methodologies useful to the farm itself, we acknowledge the diversity of ways co-production can be conceptualized. Thus, we have removed co-production, and now discuss our work as a collaborative partnership.

Minor Item:
L293 “were an intentional repository” should probably be “now constitute a useful repository” UNLESS you can prove that there was a specific intention to document behind these Hawaiian language newspapers all along. The fact that they now serve this function does not prove their original intention.

Response: We changed this to useful repository, although there is some evidence that this was an intentional documentation of stories and events for future generations.

The extensive rewrites we have undertaken of the original and revised manuscripts have streamlined, focused, and clarified the objectives and outcomes of our study of the Sumida Farm/Kalauao Spring area. At this point, we believe it will make an excellent contribution to the special issue Urban Ecosystems in PLOS ONE.

Thank you very much for your consideration.

Sincerely,

Jennifer Engels

Affiliate Faculty, Hawai‘i Institute of Geophysics and Planetology
